# A map of neurology medical residency in Brazil in 2025: really a nation?

**DOI:** 10.1055/s-0046-1827162

**Published:** 2026-08-13

**Authors:** Rairis B. Nascimento

**Affiliations:** 1 Universidade Federal de São Paulo, Escola Paulista de Medicina, Departamento de Neurologia, Núcleo de Ensino e Pesquisa em Envelhecimento Cerebral, São Paulo SP, Brazil.

**Keywords:** Neurology, Brazil, Education, Internship and Residency, Medical Residency

## Abstract

**Background:**

Neurology is a medical specialty that involves the study of various pathologies recognized for their high prevalence, morbidity and mortality. To keep up with this epidemiological scenario, we need adequately-trained professionals, whose academic training has its fundamental stage in medical residency.

**Objective:**

To characterize the existing medical residency programs (MRPs) in Neurology in Brazil in 2025, considering the availability of positions and the distribution of services throughout the country.

**Methods:**

An active search was conducted for MRP announcements for 2025 using advanced online search tools between October 2024 and March 2025. The list of medical residencies and specializations available on the website of the Brazilian Academy of Neurology (Academia Brasileira de Neurologia, ABN, in Portuguese) was used as an initial reference.

**Results:**

A total of 50 calls for applications for Neurology for 2025 were evaluated, and 332 vacancies were found, offered by 101 different programs throughout Brazil. Overall, 51.81% of the vacancies (172 positions) were concentrated in the Southeastern region, with 30.72% (102 positions) located in the state of São Paulo. In total, 5 (18,52%) Brazilian states (Acre, Amapá, Rondônia, Roraima, and Tocantins), all in the Northern region, do not offer a Neurology MRP.

**Conclusion:**

An unequal distribution of medical residency vacancies in Neurology in Brazil is evident, and this pattern converges with other realities of medicine in the country, repeating current patterns of distribution of physicians and neurologists.

## INTRODUCTION

Neurology is a medical specialty that involves the study of various pathologies recognized for their high prevalence, morbidity and mortality, such as stroke, headache, and dementia. It is estimated that approximately 1/3 of the world's population will experience some neurological disorder at some point in their lives, a figure that seems alarming considering that this burden of disability has been increasing considerably in the last 30 years. 1

To keep up with this epidemiological scenario, we need adequately-trained professionals, which becomes even more challenging in a country of continental dimensions such as Brazil. According to a Medical Demographics report published in 2025, 2 in Brazil there are 5,866 neurologists, representing 1.2% of all medical specialists in the country. In terms of the ratio between the number of professionals and the population, there are approximately 2.76 specialists for every 100 thousand inhabitants.

The academic training of these professionals, as with all specialties, has its fundamental stage in medical residency. According to the competency matrix developed by the Brazilian National Medical Residency Commission (Comissão Nacional de Residência Médica. CNRM. In Portuguese), published circa 2022, 3 upon completion of the postgraduate program in Neurology, the professional should be able to, among other skills, master the performance of general neurological examinations and cognitive screening, evaluate therapeutic approaches in various groups of neurological diseases, and interpret complementary examinations (such as electroencephalograms, electroneuromyography and others).

The objective of the present study is to characterize the existing medical residency programs (MRPs) in Neurology in Brazil in 2025, considering the availability of positions and the distribution of services throughout the country. This information aims to highlight the current scenario in the country regarding this essential stage in the professional training of these specialists and, ideally, to provide input for the continuous improvement of Brazilian medical education.

## METHODS

Between October 2024 and March 202,5 an active search was conducted for MRP announcements for 2025 on the Google (Alphabet Inc.) and Yahoo (Yahoo Inc.) search engines. The keywords used were *edital* , *residência médica* , *neurologia* and *2025* . The list of medical residencies and specializations available on the website of the Brazilian Academy of Neurology (Academia Brasileira de Neurologia, ABN, in Portuguese) was used as an initial reference. 4

During the analysis of the public notices, the total number of vacancies offered was considered, taking into account open competition and special situations (vacancies reserved for black candidates, for candidates belonging to traditional peoples, for people with disabilities, for candidates in mandatory military service, and others). The results were presented in tables and graphs, considering absolute and relative values, with standard rounding to two decimal places.

## RESULTS

The search resulted in the evaluation of 50 calls for applications for Neurology MRPs for 2025, and 332 vacancies were found, offered by 101 different programs throughout Brazil.

**Figure 1:**
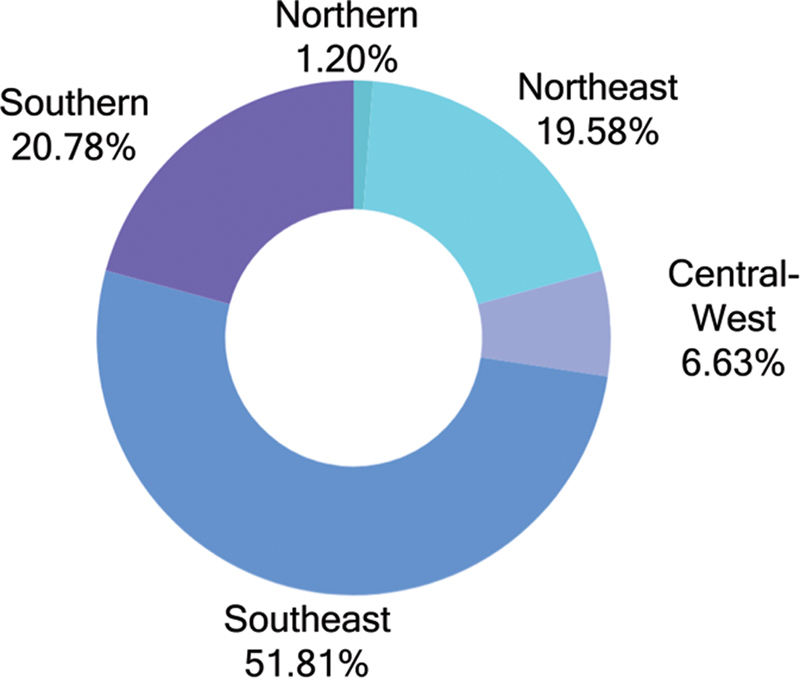
Regional distribution of medical residency vacancies offered in Neurology in Brazil in 2025.

Considering the distribution by Brazilian regions, the Southeastern region stands out with a concentration of 51.81% of the vacancies, totaling 172 positions. The second region with the highest number of vacancies is the Southern region (69 vacancies; 20.78%), followed respectively by the Northeastern region (65 vacancies; 19.58%) and the Midwestern region (22 vacancies; 6.63%). The Northern region stands out for providing only 4 (1.20%) of the vacancies ( Table 1 ) ( Figure 1 ).

**Table 1:** Distribution of the number of residency vacancies and medical residency programs in Neurology offered in Brazil in 2025

State/Region	Vacancies	%	Medical residency programs	%
Acre	0	0	0	0
Amapá	0	0	0	0
Amazonas	2	0.60	1	0.99
Pará	2	0.60	2	1.98
Rondônia	0	0	0	0
Roraima	0	0	0	0
Tocantins	0	0	0	0
**Northern region (total)**	**4**	**1.20**	**3**	**2.97**
Alagoas	5	1.51	3	2.97
Bahia	15	4.52	4	3.96
Ceará	14	4.22	2	1.98
Maranhão	1	0.30	1	0.99
Paraíba	2	0.60	1	0.99
Pernambuco	22	6.63	5	4.95
Piauí	2	0.60	1	0.99
Rio Grande do Norte	2	0.60	1	0.99
Sergipe	2	0.60	1	0.99
**Northeastern region (total)**	**65**	**19.58**	**19**	**18.81**
Distrito Federal	8	2.41	2	1.98
Goiás	8	2.41	4	3.96
Mato Grosso	2	0.60	2	1.98
Mato Grosso do Sul	4	1.20	2	1.98
**Midestern region (total)**	**22**	**6.63**	**10**	**9.90**
Espírito Santo	3	0.90	2	1.98
Minas Gerais	43	12.95	15	14.85
Rio de Janeiro	24	7.23	8	7.92
São Paulo	102	30.72	18	17.82
**Southeastern region (total)**	**172**	**51.81**	**43**	**42.57**
Paraná	29	8.73	12	11.88
Rio Grande do Sul	26	7.83	8	7.92
Santa Catarina	14	4.22	6	5.94
**Southern region (total)**	**69**	**20.78**	**26**	**25.74**
**TOTAL**	**332**	**100.00**	**101**	**100.00**

**Figure 2:**
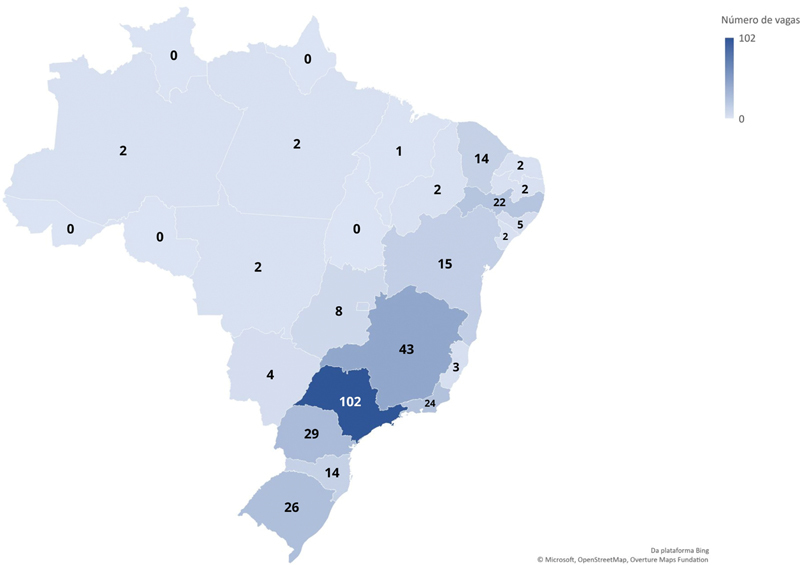
State distribution of vacancies offered in medical residency programs in Neurology in Brazil in 2025.

When analyzing the state distribution, the state of São Paulo stands out with the largest number of available positions (102 positions; 30.72%) and the greatest variety of available MRPs (18 programs; 17.82%). In second and third places respectively, considering the total number of vacancies available, are the states of Minas Gerais (43 vacancies; 12.95%) and Paraná (29 vacancies; 8.73%). The result is the same when considering the number of available MRPs, with Minas Gerais having 15 services (14.85%) and Paraná having 12 (11.88%). A total of 5 (18.52%) states (Acre, Amapá, Rondônia, Roraima, Tocantins), all in the Northern region, do not offer a Neurology MRP ( Figure 2 ).

In terms of MRPss with the largest number of available positions, the services of Hospital das Clínicas, Faculdade de Medicina de Ribeirão Preto, Universidade de São Paulo stand out, offering 14 Neurology positions in 2025, in addition to Hospital Geral de Fortaleza and Instituto de Assistência Médica ao Servidor Público Estadual de São Paulo, both offering 10 positions. Most MRPs (39 programs; 38.61%) offered 2 spots in 2025, with another 21 (20.79%) services providing 3 vacancies, these being the most common number of vacancies offered by the programs ( Table 2 ).

**Table 2:** Number of vacancies offered by medical residency programs in Neurology in Brazil in 2025

Number of vacancies offered	Number of medical residency programs	%
1	12	11.88
2	39	38.61
3	21	20.79
4	9	8.91
5	5	4.95
6	7	6.93
8	3	2.97
9	2	1.98
10	2	1.98
14	1	0.99
**TOTAL**	**101**	**100.00**

## DISCUSSION

The distribution of Neurology MRPs in 2025 proved to be quite heterogeneous across the vastness of the Brazilian territory, whether considering a regional or state perspective. Of a total of 332 medical residency vacancies offered in Neurology, the Southeastern region alone concentrates more than half (51.81%) of this amount, while in the Northern region the positions correspond to only 1.20% of the total. Similarly, the state of São Paulo had 102 vacancies (30.72%), while 5 states, corresponding to 18.52% of all federative units in Brazil, had no open positions in Neurology.

This arrangement is similar to that of other realities in Brazilian medicine. According to the most recent medical demography information published in 2025, 2 the Southeastern region concentrates 334,105 of the country's doctors (51.09%), while the Northern region retains only 4.85% (31,706 doctors) of these professionals. This same unequal distribution had already been demonstrated in the demographic data for 2020 5 (53.2% of doctors in the Southeastern region and only 4.6% in the Northern region), highlighting how little is actually being done to change this reality.

The same pattern is found when we focus the analysis on the number of neurologists in Brazil. Considering a total of 5,866 neurologists in Brazil in 2025, 2 53.5% are registered in the Southeastern region, while only 3.3% are located in the Northern region. When considering the distribution of these professionals according to the estimated total population of each region in 2025, 6 we have a ratio of approximately 3.53 neurologists for every 100 thousand inhabitants in the Southeastern region, while the ratio is of approximately 1 neurologist for every 100 thousand inhabitants in the Northernern region.

According to Scheffer et al., 7 there were a total of 947 (2.0% of the total) medical residents in Neurology in Brazil in 2024, with 326 (1.7%) of them in their first year (R1) of the program. In 2020, 5 the number of new students was 287, representing a growth of 13.59% in the last 5 years. Compared to other medical specialties, Neurology ranks 13th in terms of the total number of resident physicians. However, when considering the growth between 2018 and 2024 in the total number of R1, Neurology is only in 24th position.

In conclusion, an unequal distribution of medical residency vacancies in Neurology in Brazil is evident, whether considered from a regional or state perspective. This pattern converges with other realities of medicine in the country, repeating current patterns of distribution of physicians and neurologists. This work reinforces the importance of further research on Neurology education in Brazil and, more broadly, of reflection on medical education throughout the national territory.
